# Multidrug-resistant Tuberculosis Lymphadenitis as the Initial Presentation of Secondary Multidrug-resistant Tuberculosis: A Case Report

**DOI:** 10.7759/cureus.2363

**Published:** 2018-03-26

**Authors:** Temitayo Ogundipe, Abiodun Otolorin, Funmilola Ogundipe, Gopal Sran, Ghulam Abbas, Oladunni Filani

**Affiliations:** 1 Community and Family Medicine, Howard University Hospital; 2 Pulmonary Medicine, Howard University Hospital

**Keywords:** lymphadenitis, united states, multidrug resistant tuberculosis

## Abstract

Multidrug-resistant tuberculosis (MDR-TB) occurs when strains of Mycobacterium are resistant to the first-line anti-tuberculosis regimen. We present the case of a 22-year-old immigrant female of African descent who presented to her primary care physician complaining of a two-month history of an enlarging neck mass. Aspiration of the mass, analysis, and culture revealed colonization with a strain of Mycobacterium that was resistant to first-line anti-tuberculosis medications. She was subsequently placed on second line anti-tuberculosis medications.

## Introduction

Tuberculosis (TB) is a deadly infection of public health significance and is one of the top ten causes of death globally. In 2016, around 10.4 million people fell ill with TB with 1.7 million TB related deaths [1]. In the United States (US), there were a total of 9272 cases (2.96 cases per 100,000) in 2016 [2]. Most cases of TB respond to first line anti-tuberculosis medications. Mycobacterium TB can, however, develop resistance to any of the first line antimicrobials used to treat the infection. Multidrug-resistant tuberculosis (MDR-TB) occurs when there is at least resistance to isoniazid and rifampin, the two most potent anti-tuberculosis medications [1]. Globally in 2016, there were 490,000 cases of MDR-TB [1].

With current advancements in therapy, extra-pulmonary forms of MDR-TB are rare and are uncommon presentations of the disease; however, they appear to be on the rise [1]. There have been few cases of MDR-TB lymphadenitis presented in the literature [3-4]. We present a case of a 22-year-old human immunodeficiency virus (HIV) negative female who presented with cervical lymphadenopathy and sinus discharge at the site of the involved lymph nodes and was diagnosed with MDR-TB.

## Case presentation

A 22-year-old female of African descent who immigrated to the United States (US) seven years prior presented with a slow growing right neck mass of two months duration. There was no history of fever, cough, night sweats, fatigue, or weight loss. She reported having a positive TB quantiferon test about three years prior to current presentation due to exposure to an individual with pulmonary TB who was on treatment. At that time, a diagnosis of latent TB infection was made and she was placed on an anti-TB regimen for six months; however, she was not adherent with the therapy and stopped taking medications after one month.

**Figure 1:**
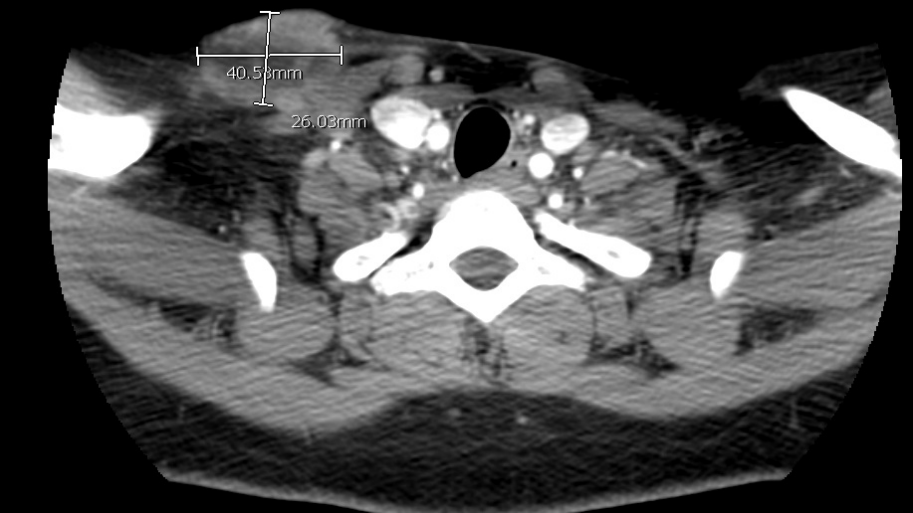
Computed tomography scan of the neck with supraclavicular mass

**Figure 2:**
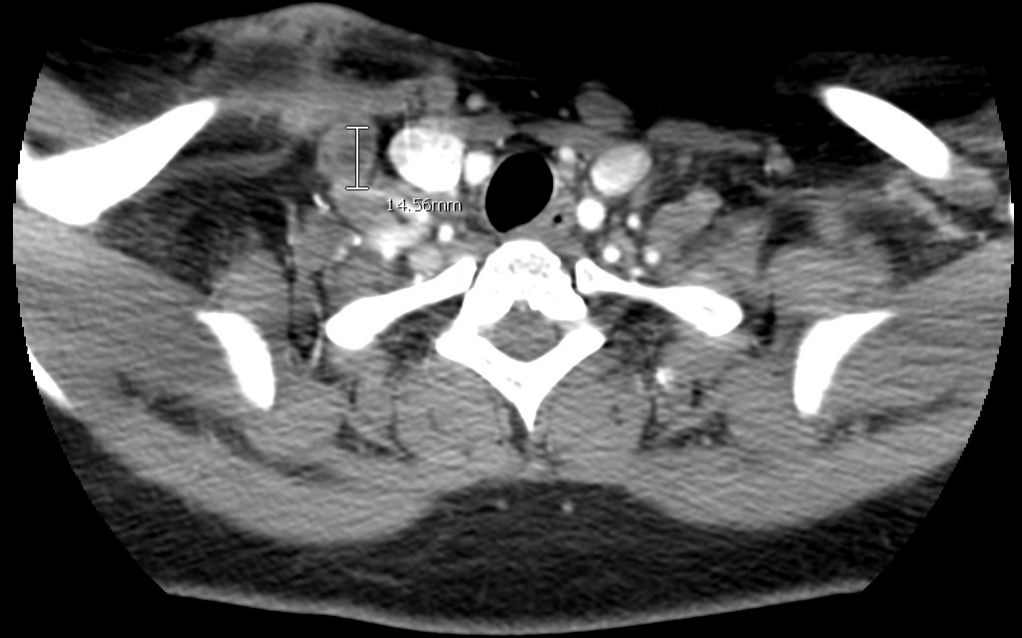
Computed tomography scan of the neck with supraclavicular lymph node

She presented three years after exposure with a neck mass. On examination, she had a 45 x 35 mm soft tissue neck mass that was mobile, soft, and tender. There were no palpable enlarged lymph node groups in the neck or any other part of the body. Systemic examination of the body was essentially normal. Laboratory examination revealed hemoglobin of 12.1 g/dl, lymphocyte count of 2.2 x10e3/ul, random blood glucose of 110 mg/dl, BUN of 4 mg/dl and creatinine of 0.44 mg/dl. All other laboratory parameters were within normal limits. Hepatitis panel was negative (hepatitis B & C) and HIV was negative for both HIV 1 and 2. Ultrasound of the neck showed solid lobulated masses in the right supraclavicular region concerning for abnormal lymph nodes. A follow up computed tomography (CT) scan of the neck showed a 19 x 40.58 x 26.03 mm non-homogenous supraclavicular mass with indistinct borders and stranding of adjacent fat, and a 15 mm non-homogenous right supraclavicular lymph node (Figures 1-2).

**Figure 3:**
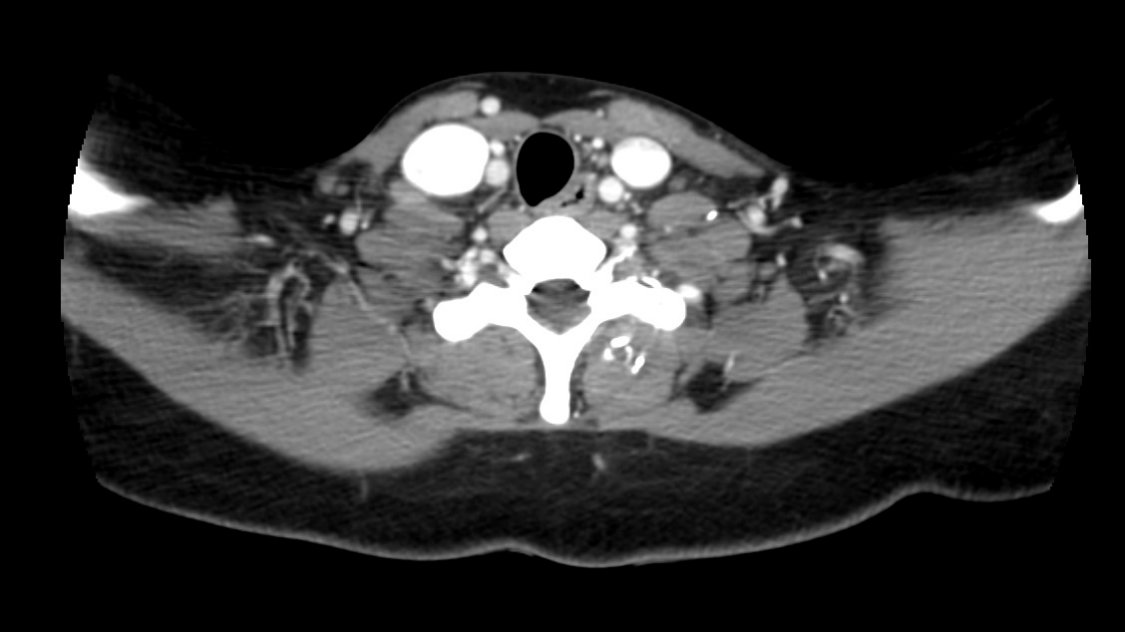
Computed tomography scan of the neck one year after initiation of anti-tuberculosis therapy

Pulmonary involvement was ruled out with a bronchoscopy with bronchoalveolar lavage which came back acid-fast bacilli (AFB) negative and AFB polymerase chain reaction (PCR) negative. A CT scan of the chest was obtained and it showed no infiltrates. The TB quantiferon assay was positive; subsequently, fluid was aspirated from the neck mass and sent for fluid analysis (culture, gram stain, AFB stain, and TB PCR). AFB culture of the aspirate was positive and it was isolated from the broth culture. The organism was resistant to streptomycin, isoniazid, and rifampin but susceptible to ethambutol and pyrazinamide. She was diagnosed with MDR-TB lymphadenitis and was started on ethambutol, pyrazinamide, moxifloxacin, amikacin, linezolid, and cycloserine. A second line drug resistance testing was also obtained which showed resistance to ethambutol, and was then switched to a susceptible alternative - ethionamide. She was subsequently referred to the local department of health, the infectious disease unit, and her primary care physician for close follow- up and was scheduled to undergo therapy for two years. Repeat CT scan soft tissue of the neck obtained one year after initiation of therapy showed complete resolution of the mass (Figure 3).

## Discussion

TB is a deadly mycobacterial infection spread via airborne droplets from infected persons; it remains a significant cause of morbidity and mortality worldwide. It afflicts a quarter of the world population as latent TB [5]. MDR-TB is an infection by a strain of mycobacteria tuberculosis resistant to at least isoniazid and rifampin; two widely used potent anti-tuberculosis medication. MDR-TB arises either as a result of direct infection from a person with a drug-resistant strain (primary resistance) or poor compliance with strict anti-tuberculosis treatment regimen (secondary or acquired resistance) [6].

In 2016, there were about 6.1 million new cases of TB worldwide with 4.1 percent due to MDR-TB [1]. In 2016, in the US, there were 9272 reported cases of TB with 96 cases of MDR-TB [7]. Compared to the global burden, cases of MDR-TB are relatively rare in the US with most (91%) of the MDR-TB cases occurring in foreign-born persons [7]. Cervical lymphadenitis is an extra-pulmonary manifestation of TB. MDR-TB lymphadenitis is a rare manifestation of a disease of significant public health concern. While there have been reported cases of MDR-TB lymphadenitis in TB endemic regions of the world [3-4]; cases of MDR-TB lymphadenitis represent an unusual and atypical presentation of the disease in the US.

The cost of treatment places a major strain on healthcare systems with the cost of treatment for TB and MDR-TB in the US estimated at $46,000 and $294,000 respectively; a significant cost often borne by the public health system [8]. This serves as an impetus for effective control. With a comprehensive strategic framework, the US has made significant strides towards its national goal of elimination of the disease, defined as achieving a case rate of less than one case per million populations. In 2016, TB incidence was 2.9 per 100,000 cases compared with 3.4 per 100,000 cases in 2015 that is in keeping with an annual sustained decline in TB incidence rate over two decades [9]; however, it remains above the national goal of elimination. Current epidemiologic models suggest that even if the rate of previous annual declines were sustained, TB elimination will not occur by the end of this century [10].

With the advent of increased globalization, immigration, and refugee resettlement, cases of TB and drug-resistant TB may continue to rise and represent a threat to current control programs. There may be a need to create and implement policies that intensify disease surveillance efforts, particularly in populations at increased risk. Public health systems may also need to be strengthened to ensure that patients on anti-tuberculosis medications are closely monitored to prevent the development of resistant forms of the disease, which was what may have occurred in our case.

## Conclusions

In conclusion, drug-resistant tuberculosis has widespread health, social, and economic ramifications and its emergence threaten gains already made at national and global levels in TB care and control. There must be a sustained effort by healthcare professionals to work together to support the global vision of a world free of TB.
